# New onset of type 2 diabetes as a complication after cancer diagnosis: A systematic review

**DOI:** 10.1002/cam4.3666

**Published:** 2020-12-23

**Authors:** Ara Jo, Lisa Scarton, LaToya J. O'Neal, Samantha Larson, Nancy Schafer, Thomas J. George, Juan M. Munoz Pena

**Affiliations:** ^1^ Department of Health Services Research, Management and Policy University of Florida Gainesville FL USA; ^2^ Department of Family Community and Health Systems Science College of Nursing University of Florida Gainesville FL USA; ^3^ Department of Family, Youth and Community Sciences Institute of Food and Agricultural Sciences University of Florida Gainesville FL USA; ^4^ Health Science Library University of Florida Gainesville FL USA; ^5^ Division of Hematology and Oncology College of Medicine University of Florida Gainesville FL USA; ^6^ Division of Endocrinology, Diabetes, and Metabolism College of Medicine University of Florida Gainesville FL USA

**Keywords:** breast cancer, cancer management, digestive cancer, epidemiology and prevention, gastric cancer, survival

## Abstract

**Background**

Despite improved survival rates, cancer survivors are experiencing worse health outcomes with complications of treatment, such as type 2 diabetes mellitus (T2D), that may deteriorate survivorship. The purpose of this review was to provide a comprehensive review of T2D incidence following cancer diagnosis. Methods: The study included: (1) cohort studies, (2) cancer diagnosis by a doctor, (3) incidence of T2D after diagnosis of cancer, and (4) adult patients over 18 years. Studies that focused on patients who had T2D as a preexisting condition at cancer diagnosis were excluded. Results: Of a total of 16 studies, overall incidence of T2D ranged from 5.4% to 55.3%. The highest T2D incidence rate was observed in colorectal patients with cancer (53%). While results in prostate patients with cancer were mixed, patients who underwent androgen deprivation therapy (ADT) had a significantly higher incidence of new‐onset T2D (12.8%, *p* = 0.01). Patients treated with chemotherapy within 1–5 years of initial diagnosis of colorectal cancer were at approximately 30% higher risk of T2D. One study found that 48% of T2D was preventable with optimal management during the process of patient care. Conclusion: Blood glucose management may allow physicians to intervene early and improve outcomes among patients with cancer.

## Graphical abstract

About half of patients with cancer reported new onset of type 2 diabetes after cancer diagnosis and treatment. Forty‐eight percent of type 2 diabetes incidence was preventable with optimal management during the process of patient care.

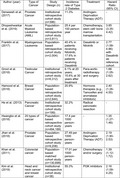

## 1 INTRODUCTION

Cancer is the second leading cause of death within the United States.^1^ With advanced treatment and cancer screening provisions, the 5‐year survival of all cancer types increased to 67.1% in 2015.^2^ In 2016, more than 15.5 million Americans were patients living with cancer or survivors, a number projected to increase to more than 20.3 million by 2026.^3^ Despite this increase in survival rates, cancer survivors suffer worse outcomes than previously noted, such as experiencing poor quality of life, depressive symptoms, and physical inactivity.^2^ More cancer complications have been detected among survivors in recent years, attenuating survival rates.^4^ Several long‐term complications in patients with cancer impact functional return to a high quality of life including hernia, cardiovascular diseases, intestinal obstruction, and urinary retention.^5^, ^6^

Type 2 diabetes (T2D) is strongly associated with the incidence of cancer. A large body of epidemiological studies report that individuals who have T2D are at an increased risk of breast cancer and prostate cancer, among others.^7^, ^8^ Further, the concurrence of T2D and cancer has accelerated mortality in patients.^8^ Despite this knowledge, little is known about the relationship between T2D and cancer, the development and diagnosis of T2D after cancer diagnosis, and/or treatment. Thus, the aim of this study was to provide a comprehensive review of T2D incidence among diverse patients with cancer who underwent disease‐specific treatment.

## 2 METHODS

The systematic review was conducted in accordance with PRISMA guidelines.^9^ A primary objective was to identify the incidence of type 2 diabetes after a cancer diagnosis regardless of cancer types. Secondary objective was to identify preexisting clinical conditions and to report risk of new‐onset type 2 diabetes stratified by types of treatment. PubMed, CINAHL, Web of Science, and EMBASE were used for search by a librarian. Literature published between 2000 and 2019 were included. For this systematic review study, ethical approval was waived by the Institutional Review Board.

### 2.1 Study selection

**FIGURE 1:**
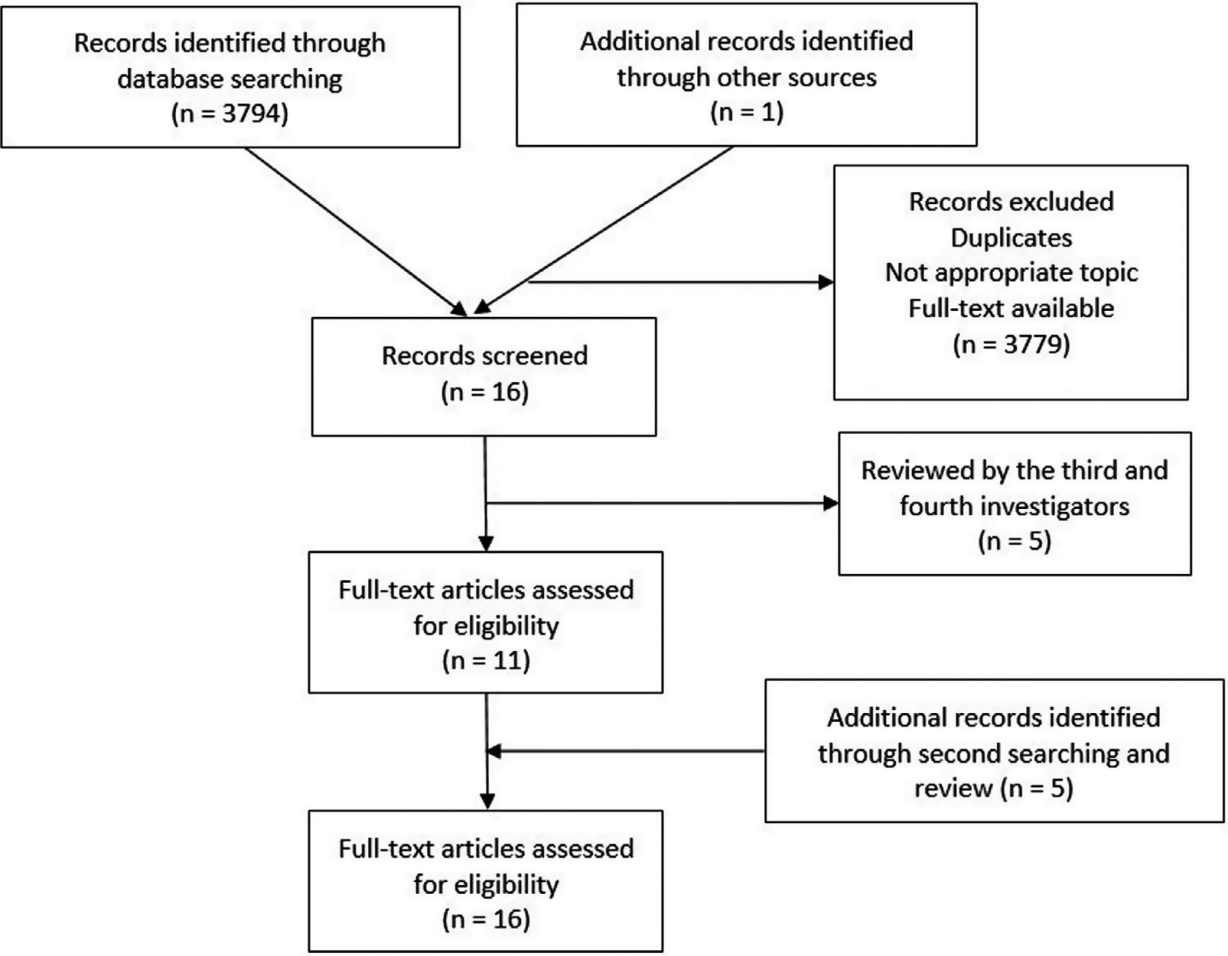
Flow of PRISMA guideline

Criteria for inclusion in the analysis were: (1) cohort studies using longitudinal data, (2) cancer diagnosis by a health‐care provider, (3) incidence of T2D after diagnosis of cancer, (4) adult patients over 18 years of age, (5) English language study published in scientific journals, (6) full‐text available, and (7) completed study. Review papers, protocols, commentaries, and randomized clinical trials that are not able to track onset of T2D were excluded. Additionally, studies that focused on patients who had T2D as a preexisting condition at cancer diagnosis were excluded. Two independent investigators reviewed title, abstract, and full texts and disagreements were settled by the two additional investigators. Thus, the final included studies were selected based upon agreement between four independent investigators (Figure 1).

### 2.2 Outcome

A primary outcome was incidence of T2D that is defined by the World Health Organization or American Diabetes Association after diagnosis of cancer.^10^, ^11^ Diagnosis of T2D by a physician on electronic health record using ICD‐9 codes was also eligible. Type 1 diabetes and other form of diabetes, including gestational diabetes, were excluded due to different physiological mechanism.

## 3 RESULTS

### 3.1 Study characteristics

Of the total 3795 articles, 16 met all inclusion criteria. Published studies were conducted between 2007 and 2019 in the following eight countries: Canada, China, Israel, Netherlands, South Korea, Taiwan, UK, and USA. Included studies comprised from 1 to 20 different types of cancer. A complete list of cancer types is displayed in Table 1. Six studies used a population‐based database,^12^, ^13^, ^14^, ^15^, ^16^, ^17^ while the remaining studies used individual hospital medical records.^18^, ^19^, ^20^, ^21^, ^22^, ^23^, ^24^, ^25^, ^26^, ^27^ The age of the study population ranged from 20 to 105 years old. Two study reported the proportion of race/ethnicity among patients,^18^, ^25^ while three studies highlighted patient socioeconomic status (SES) (i.e., education, immigration status, household income, and employment).^13^, ^15^, ^19^ Consistent with national trends, assessment of patient demographics revealed that those with less education and lower income exhibit higher T2D incidence. Three studies presented lifestyle patterns of patients, such as physical activity, healthy diet,^19^ and smoking.^18^, ^23^ Among study patients, those who had an unhealthy lifestyle had a higher incidence of T2D.^19^, ^23^ Additionally, Hamood and colleagues reported health‐care utilization and found that higher frequency of outpatient visits was associated with T2D incidence in patients with cancer.^21^

**TABLE 1:** Summary of selected studies

Author (year)	Type of cancer	Study design (n)	Incidence rate of Type 2 diabetes	Treatment	Hazard ratio (95% CI)
Derweesh et al. (2017)	Prostate cancer	Institutional retrospective cohort study (n = 396)	11.3%	Androgen deprivation therapy (ADT)	‐
Dhopeshwarkar et al. (2019)	Acute myeloid leukemia (AML)	Population‐based retrospective cohort study (n = 3,911)	25.4 per 100 person year	Chemotherapy, stem cell/ bone marrow transplantation	3.85 (3.35–4.42)
Franklin et al. (2017)	Myelogenous leukemia	Population‐based retrospective cohort study (n = 2,004)	40.4% in patients receiving Nilotinib, 17.6% in patients receiving Dasatinib	Dasatinib or Nilotinib	2.77 (1.58–4.86) with Dasatinib as reference cohort
Groot et al. (2018)	Testicular cancer	Institutional retrospective cohort study (n = 6,312)	6.1% at 20 years; 15.6% at 30 years after treatment	Para‐aortic radiotherapy and surgery	1.66 (1.05–2.62)
Hamood et al. (2018)	Breast cancer	Population‐based retrospective cohort study (n = 2,644)	20.9%	Hormone therapies (e.g., Tamoxifen and aromatase inhibitors)	2.40 (1.26–4.55)
He et al. (2013)	Pancreatic cancer	Institutional retrospective cohort study (n = 199)	32.2%	Radical pancreatic resection	‐
Hwangbo et al. (2018)	20 types of cancer	Population‐based retrospective cohort study (n = 494,189)	17.4 per 1000 person‐years	‐	1.35 (1.27–1.46)
Jhan et al. (2018)	Prostate cancer	Population‐based retrospective cohort study (n = 9,208)	27.49 per 1000 person‐years	Androgen Deprivation Therapy (ADT)	2.19 (1.90–2.53)
Khan et al. (2011)	Colorectal cancer	Population‐based matched cohort study (n = 130,699)	17.01 per 1000 person‐years	Chemotherapy and/or surgery	1.39 (1.12–1.72)
Kim et al. (2019)	Head and neck cancer and breast cancer	Institutional retrospective cohort study (n = 38)	55.3%	PI3K inhibitors	2.16 (1.09–4.25)
Lipscombe et al. (2013)	Breast cancer	Population‐based retrospective cohort study (n = 24,976)	9.8%	Intravenous adjuvant chemotherapy	1.21 (1.09–1.35)
Li et al. (2015)	Pancreatic cancer	Institutional retrospective case‐control cohort study (n = 1,328)	15.8%	‐	1.23 (1.09–1.40)
Lu et al. (2014)	Breast cancer	Institutional retrospective cohort study (n = 128)	17.2%	Surgery and/or chemotherapy	‐
Singh et al. (2016)	Colorectal cancer	Population‐based retrospective cohort study (n=)	53%	Chemotherapy	1.53 (1.42–1.64) in year 1 1.19 (1.05–1.35) in year 5
Teoh et al. (2015)	Prostate cancer	Institutional retrospective cohort study (n = 388)	12.8%	Androgen deprivation therapy (ADT)	3.34 (1.19–9.39)
Yang et al. (2015)	Breast cancer	Population retrospective cohort study (n = 31,112)	8.36%	Morphine therapy	1.24 (1.04–1.49)

### 3.2 Incidence of type 2 diabetes

Comprehensive T2D incidence in patients diagnosed with one of the 20 investigated cancer types is 17.4 per 1000 person‐years.^13^ Overall incidence of T2D ranged from 5.4% to 55.3% based on nine studies. The highest incidence rate was observed in colorectal cancer patients (53%).^16^ The hazard ratio (HR) of T2D development was highest among patients diagnosed with pancreatic cancer (HR: 5.15, 95% CI: 3.32–7.99).^13^ This was followed by the HR of acute myeloid leukemia (AML) patients, 3.85 (95% CI: 3.35–4.42).^19^ Four studies evaluated T2D incidence at different time periods following diagnosis.^13^, ^15^, ^16^, ^20^ Two of these four studies found that, in the first year of follow‐up since diagnosis, the HRs of T2D development were highest for colorectal cancer patients, at 1.53,^16^ and 1.47 (95% CI: 1.35–1.60) overall for patients with cancer.^13^ Groot and colleagues reported the long‐term incidence rate of T2D at 20 and 30 years after testicular cancer treatment and found that the longer cancer survivors live, the higher the incidence of T2D.^20^ Lipscombe and colleagues found the similar pattern that displays the increase in cumulative incidence over time in patients with breast cancer.^15^

### 3.3 Preexisting conditions

Multiple comorbidities, such as hypercholesterolemia,^20^ hypertension,^14^, ^16^, ^20^, ^25^ cardiovascular disease,^14^, ^15^, ^16^, ^19^, ^25^, ^27^ dyslipidemia,^14^, ^27^ dementia, liver disease, renal disease, rheumatologic disease, peptic ulcer disease, HIV/AIDS,^12^, ^15^, ^27^ weight loss,^25^ depression,^14^ and chronic kidney disease,^12^, ^14^, ^15^ at diagnosis were considered. Those with new onset of T2D were more likely to have at least one chronic disease.^16^ Most studies took into account BMI, with mixed effects reported. BMI category did not show a consistent significant role in T2D incidence,^24^ whereas obesity, defined by BMI of 30 kg/m^2^, was significantly associated with T2D incidence.^18^, ^20^, ^21^, ^26^ The study of Li and colleagues included BMI in statistical model, whereas it did not address whether BMI was a significant factor or not.^25^

### 3.4 Treatments and risk of T2D development

#### 3.4.1 Breast cancer (n = 6)

Overall incidence of T2D in patients with breast cancer ranged from 9.7% to 20.9%. Overall HR of T2D incidence among breast cancer was 1.60 (95% CI: 1.27–2.01) regardless of treatment.^13^ Its incidence varied across types of treatment. With direct risk of treatment, Lu and colleagues reported that 17.2% of patients developed T2D after 3 months of chemotherapy and/or surgery.^26^ Lipscombe and colleagues found that postmenopausal women with breast cancer showed significantly higher incidence of T2D after 2 years of diagnosis (HR: 1.21, 95% CI: 1.09–1.35). Moreover, patients who underwent adjuvant chemotherapy reported significantly higher risk of T2D (HR: 1.24, 95% CI: 1.12–1.38), while its risk was decreased afterward.^15^ Yang and colleagues identified that users of morphine therapy were 1.24 times more likely to suffer from T2D than non‐morphine users (95% CI: 1.04–1.49). Further, they identified that the risk of T2D increased with increased morphine dose by every 20 DDD (HR = 1.02, 95% CI: 1.00–1.03), age, and Charlson Comorbidity Index.^17^ With indirect risk of treatment, Kim and colleagues reported comprehensive HR of T2D incidence when PI3K inhibitor was used for breast cancer and head and neck cancer.^24^ Patients who underwent hormone therapy were associated with an increased risk of T2D development compared to patients not receiving hormone therapy (HR: 2.40 95% CI: 1.26–4.55), while Aromatase inhibitors were associated with the highest risk of T2D development (HR: 4.27, 95%CI 1.42–12.84).^21^ In addition, the duration of hormone therapy was associated with an increased risk of T2D. Patients who underwent hormone therapy for longer than 1 year were at 6.5 times higher risk of T2D incidence than those without therapy (95% CI: 1.84–22.84).^21^ The final study did not report the risk of T2D due to non‐significant results.^23^

#### 3.4.2 Colorectal cancer (n = 2)

A Canadian study reported a significant association of T2D development risk among the 53% of patients.^16^ Patients treated with chemotherapy within 1–5 years of initial diagnosis were at an approximately 30% higher risk of T2D when compared to patients who did not receive chemotherapy. Between year 2 and year 3, the HR of T2D was found to be the highest (HR: 1.32, 95% CI: 1.15–1.52).^16^ A British study reported similar significant findings, with the HR of T2D development posttreatment with either chemotherapy or surgery described in Table 1.^23^

#### 3.4.3 Pancreatic cancer (n = 3)

Three studies examined T2D incidence among patients with pancreatic cancer. Hwangbo and colleagues reported the highest HR of T2D (HR: 5.15, 95% CI: 3.32–7.99), regardless of treatment option.^13^ The second study assessed T2D as a surgical complication of radical pancreatic resection. While it did not provide a HR, 32.2% of patients developed T2D within 2 years of undergoing radical pancreatic resection. With respect to overall survival, those with new onset of T2D had longer survival, although this was not significantly different from those patients who had long‐standing T2D after resection (*p* = 0.17).^22^ Li and colleague found that metastatic patients with new‐onset T2D showed significantly higher HR of death compared to patients without T2D (HR: 1.35, 95% CI: 1.11–1.63) regardless of treatment option.^25^

#### 3.4.4 Prostate cancer (n = 4)

T2D incidence and risk among patients with prostate cancer was examined in four studies. Hwangbo and colleagues reported no significant increase in risk of T2D among patients with prostate cancer.^13^ Three other studies examined T2D incidence ranging from 11.3% to 12.8% among patients undergoing androgen deprivation therapy (ADT).^14^, ^18^, ^27^ Jhan and colleagues reported significantly higher incidence of T2D in patients who underwent ADT (27.49 person‐years) compared to patients without ADT.^14^ Two studies reported significantly higher risk of new‐onset T2D in patients who underwent ADT (HR: 3.34, 95% CI: 1.19–9.39, *p* < 0.01 and HR: 2.19, 95% CI: 1.90–2.53).^14^, ^27^

#### 3.4.5 Testicular cancer (n = 1)

Only one study examined T2D incidence among patients with testicular cancer (Table 1). Groot and colleagues found that the HR of T2D development in patients who underwent para‐aortic radiation was 1.66 (95% CI: 1.05–2.62).^20^ This study, specifically, examined T2D incidence in patients stratified by radiation field and radiotherapy dose. Variation in radiation field did not show significantly higher risk.

#### 3.4.6 Acute myeloid leukemia (AML) (n = 1)

Only one study reported T2D incidence and risk among patients with AML. AML patients showed increased T2D risk regardless of treatment options (HR: 3.85, 95% CI: 3.35–4.42).^19^

#### 3.4.7 Chronic myelogenous leukemia (CML) (n = 1)

Only one study examined the incidence of T2D and risk among patients diagnosed with CML. Franklin and colleagues conducted a retrospective observational cohort study of CML patients treated with either dasatinib and nilotinib. The incidence of T2D was found to be higher in patients receiving nilotinib than those receiving dasatinib at 40.4% (95% CI: 27.6–57.0) and 17.6% (95% CI: 11.1–28.4), respectively. When dasatinib was used as a reference cohort, it was found that patients receiving nilotinib were significantly more likely to develop T2D (HR 2.77, 95% CI: 1.58–4.86, *p* = 0.0004).^12^

#### 3.4.8 Head and neck cancer (n = 2)

Kim and colleagues examined the overall HR of T2D among head and neck and breast cancer patients undergoing treatment with PI3K inhibitor.^24^ Patients with newly diagnosed T2D presented with a significantly higher HbA1c (7.3 ± 1.1% vs. 6.0 ± 0.3% *p* < .01) during PI3K inhibitor treatment. Particularly, persistent T2D group presented much higher fasting glucose level (180.1 mg/dL vs. 100.8 mg/dL, *p* < .01) compared to those with T2D remission during treatment. However, Hwangbo and colleagues did not find a significant association between oral, lip, pharynx, and esophagus cancers and T2D incidence.^13^

## 4 DISCUSSION

This review study found a substantial incidence of T2D discovered in patients who had been diagnosed with a variety of cancer types. Such results may be, in large part, due to adverse effects of cancer treatments. Particularly, 48% of T2D incidence is deemed preventable with optimal management of glucose during the process of patient care.^21^ This study indicates that structured T2D prevention protocols for patients with cancer may reduce the risk of new onset of T2D and other tangential adverse outcomes. To our knowledge, this is the first study to generate a comprehensive review of the incidence of new onset of T2D among patients with diverse cancers.

Increased risk of T2D incidence was detected among patients diagnosed with multiple forms of cancer. Regardless of systemic treatment type, the risk of T2D development was increased during follow‐up years among patients with colorectal and pancreatic cancer.^13^, ^16^ One possible reason for this is the alteration of glucose metabolism that follows cancer diagnosis. Glucose metabolism alteration was commonly observed in patients with cancer due to the hypoxic conditions.^28^ Additionally, corticosteroids are commonly used in tandem with chemotherapy, either as a primary component of treatment or as an adjunct to treat drug reactions or nausea. Existing literature identifies corticosteroid use as significantly associated with hyperglycemia, indicating that it may cause T2D.^29^, ^30^ This is noteworthy given that either high or low doses of corticosteroid may alter glucose level in a healthy population, therefore the effect could exacerbate T2D risk in patients with cancer.^31^ Other cancer therapies (e.g., phosphatidylinositol 3‐kinase inhibition) can lead to metabolic alterations, hyperglycemia, and subsequent T2D development.^32^

Obesity, defined by BMI of greater than 30 kg/m^2^, was a significant predictor of T2D incidence in diverse cancer types. Some patients with breast cancer experienced significant weight gain with hormone therapy and adjuvant chemotherapy.^33^, ^34^ In addition, patients with prostate cancer who underwent ADT reported weight gain of 3.9%, particularly in abdomen.^35^ This may be a similar mechanism surrounding women with breast cancer treated with hormonal therapy who develop T2D. In both situations and in both sexes, hormonal manipulation can induce a menopausal state, the latter of which is known to be associated with T2D development in non‐cancer patients.^36^ Physical activity reduction, hyperphagia, and changes in thermogenesis are potential consequences of cancer treatments. With worsened physical function, subsequent weight gain and resulting obesity could accelerate development of T2D. Thus, appropriate lifestyle changes may prevent such downstream complications. Additionally, cancer cachexia or treatment‐associated metabolic changes can reduce muscle mass while maintaining relative weight. This adiposity replacement phenomenon is otherwise known as sarcopenic obesity and is associated with increased leptin levels and insulin resistance.^37^ Thus, body composition and loss of lean muscle mass, rather than absolute weight gain, may be a more important risk factor for cancer‐associated T2D development.

Glucose assessment includes selected preoperative or postoperative glucose testing for patients with cancer. Perioperative or postoperative hyperglycemia was associated with high mortality, complications, and surgical site infection in patients with cancer or patients undergoing general surgery.^38^, ^39^ However, despite protocol, the decision to delay cancer treatment or provide care for patients with high glucose may be judicious for physicians and is largely based on their experience and level of knowledge regarding the risk of glucose imbalance during treatment. As such, there is an explicit need for standardized protocols for preoperative, perioperative, and postoperative blood glucose assessment in patients receiving treatment for various forms of cancer. These types of protocols would act to mitigate the risk of T2D development and reduce mortality.

## 5 LIMITATION

This study has limitations. First, due to the variation in cancer types, this study was not able to produce an aggregate incidence rate of T2D among patients diagnosed with cancer. Second, most of the included studies utilized individual hospital medical records which may both overestimate T2D incidence rate, given the frequency of glucose monitoring, and underestimate T2D incidence rate, given that the majority of longitudinal cancer care is provided in an ambulatory environment. Moreover, standard of diagnosis for diabetes may not be uniform across the study as this study included international studies. Future study may focus on the study using standardized diagnosis method and produce more accurate incidence. Besides, a causal relationship between T2D incidence and cancer may not be established. Some studies were limited to assessing T2D incidence after the implementation of some form of cancer treatment, therefore these treatments may disrupt the true etiology of T2D development among newly diagnosed patients with cancer. As a result, further studies exploring the physiological mechanism of T2D incidence in patients recently diagnosed with cancer are needed. Lastly, there may be a detection bias. As patients with cancer are likely to have regular check‐up and more health‐care utilization,^40^ early detection of type 2 diabetes may increase incidence compared to other diseases without regular check‐up.

## 6 CONCLUSION

Continuous and long‐term monitoring of blood glucose may detect T2D in early stage disease before organ damage occurs in patients with cancer. Prevention measures for elevated glucose allow physicians to prevent unpredictable T2D incidence during cancer treatment. Patients with digestive system‐related cancers should receive particular attention when protocols to control glucose level are developed.

## CONFLICT OF INTEREST

The authors declare no potential conflict of interest.

## FUNDING INFORMATION

None.

## Data Availability

Data sharing is not applicable to this article as no datasets were generated or analyzed during the current study.
